# A noninferiority cluster randomised evaluation of a broflanilide indoor residual spraying insecticide, VECTRON T500, for malaria vector control in Tanzania

**DOI:** 10.1038/s41598-025-99809-9

**Published:** 2025-04-29

**Authors:** Njelembo J. Mbewe, Patrick K. Tungu, Louisa A. Messenger, John Bradley, Peter E. Mangesho, Boniface Shirima, Oliver Moshi, Magreth F. Shayo, Mohammed Seif, Natalie M. Portwood, Janneke Snetselaar, Salum Azizi, Frank Magogo, Peter Mabenga, Wema Sudi, George Mlay, Matthew J. Kirby, Franklin W. Mosha, William Kisinza, Johnson Matowo, Graham Small, Mark W. Rowland

**Affiliations:** 1 Department of Disease Control, London School of Hygiene and Tropical Medicine, London, WC1E7HT UK; 2 Department of Parasitology, Kilimanjaro Christian Medical University College, Moshi, Tanzania; 3 Pan-African Malaria Vector Research Consortium PAMVERC, Muheza & Moshi, Tanzania; 4 National Institute for Medical Research, Muheza, Tanzania; 5 Parasitology and Vector Biology (PARAVEC) Laboratory, School of Public Health, University of Nevada, Las Vegas, Las Vegas, NV USA; 6 Innovative Vector Control Consortium, Liverpool, UK

**Keywords:** Randomized controlled trials, Malaria, Entomology, Epidemiology

## Abstract

Effective malaria vector control is being undermined by the rapid spread of insecticide resistance. VECTRON T500, a new indoor residual spraying (IRS) product containing the active ingredient broflanilide as a 50% wettable powder (WP), was previously shown to be efficacious in experimental hut trials. A two-arm non-inferiority cluster randomized controlled community trial was conducted in Muheza District, Tanga Region, Tanzania. VECTRON T500 was compared to the IRS product Fludora Fusion (clothianidin 50% + deltamethrin 6.25% WP-SB). Sixteen village clusters were pair-matched on baseline vector densities and allocated to reference and intervention arms. Monthly CDC light trapping sampled mosquitoes to estimate vector density, indoor biting, sporozoite and entomological inoculation rate (EIR). The non-inferiority margin of mosquito density was defined as a density ratio of 1.5. Susceptibility to IRS active ingredients was assessed in one of the local vectors, *Anopheles gambiae* sensu lato (s.l.), using WHO/CDC bottle bioassays. The residual efficacy of both IRS products was monitored for 12 months using susceptible and pyrethroid resistant *An. gambiae* sensu stricto (s.s.) mosquitoes. This study is registered with ClinicalTrials.gov (NCT05150808). A total of 916 and 844 houses were sprayed with Fludora Fusion and VECTRON T500, respectively, with equitable spray coverage. *An. gambiae* s.l. was resistant to deltamethrin but susceptible to clothianidin and broflanilide. The density ratio adjusted for baseline Anopheline mosquito density was 0.77 (95% CI: 0.45–1.29). The baseline adjusted sporozoite rate and EIR differences between the two trial arms were 0.84% and 15.61%, respectively. The residual efficacy was > 80% mortality for VECTRON T500 and Fludora Fusion, on both mud and concrete walls, 12 months post spraying. VECTRON T500 was non-inferior to Fludora Fusion in terms of its ability to reduce vector density, sporozoite rate and EIR, providing an additional vector control tool with a new mode of action for malaria prevention and insecticide resistance management.

**Supplementary Information:**

The online version contains supplementary material available at 10.1038/s41598-025-99809-9.

## Introduction

Insecticide-treated nets (ITNs) and indoor residual spraying (IRS) remain important components in all malaria vector control programmes^1^. Between 2000 and 2015, ITNs and IRS contributed to the decline in malaria cases by 68% and 10%, respectively^2^. In 2021, about 220 million ITNs were delivered to malaria endemic countries, 10 million fewer than in 2020^3^. Whilst the delivery of ITNs by manufacturers to endemic countries in sub-Saharan Africa increased from 145 million in 2010 to about 176 million in 2021, there was a decline in the number of people at risk protected by IRS in sub-Saharan Africa from 153 million to 80 million during the same period^3^. Several factors, including widespread development of insecticide resistance, lack of alternative insecticides and limited resources, and disruptions caused by the COVID-19 pandemic, have been attributed to this decrease in IRS coverage^4^.

The Global Plan for Insecticide Resistance Management (GPIRM) proposed the development of new active ingredients (AIs) with different modes of action to support strategies for insecticide resistance management^1^. These resistance management strategies focus on IRS and propose using mixtures and rotations of insecticides from different chemical classes^1^. This has stimulated the development of novel IRS products containing new classes of insecticides by private sector manufacturers^5–8^.

VECTRON™ T500, a newly developed IRS product containing the active ingredient broflanilide (trade name TENEBENAL™) was recently listed by the World Health Organization Prequalification Vector Control Product Assessment Team (WHO PQT/VCP)^9^. Broflanilide is a meta-diamide insecticide which is classified by the Insecticide Resistance Action Committee (IRAC) in Group 30, γ-aminobutyric acid gated chloride channel allosteric modulators^10,11^. It has previously been shown to be efficacious against both insecticide susceptible and pyrethroid resistant *Anopheles*mosquitoes in small-scale experimental hut trials^7,8,12^. It has also demonstrated long residual activity and was efficacious against *Anopheles (An.) coluzzii* and *An. gambiae *s.s. populations in a community trial in Benin^13^.

Here, to inform the evaluation of VECTRON™ T500 by WHO PQT/VCP, this IRS product was evaluated for non-inferiority compared to the WHO PQT/VCP listed IRS product Fludora^®^ Fusion (Envu™) in a community-level trial with entomological endpoints. Its residual activity and effect on the malaria entomological inoculation rate (EIR) and other transmission endpoints were also assessed.

## Results

The number of houses per study cluster ranged between 100 and 200 houses. In most study clusters, houses with walls made of mud were predominant, i.e. 80.75% (95% CI: 75.0–86.5%) in the Fludora^®^ Fusion and 77.6% (95% CI: 65.2–90.0%) VECTRON™ T500 arms. Coverage of ITNs in study clusters were 85.0% (95% CI: 73.2–96.9%) and 90.8% (95% CI: 81.1–100%) in the Fludora^®^ Fusion and VECTRON™ T500 arms, respectively.

Between February-March 2021, a total of 844 houses were sprayed in the intervention arm and 916 houses in the reference arm. The community accepted the intervention and did not report adverse effects.

### Residual bioefficacy

**Fig. 1:**
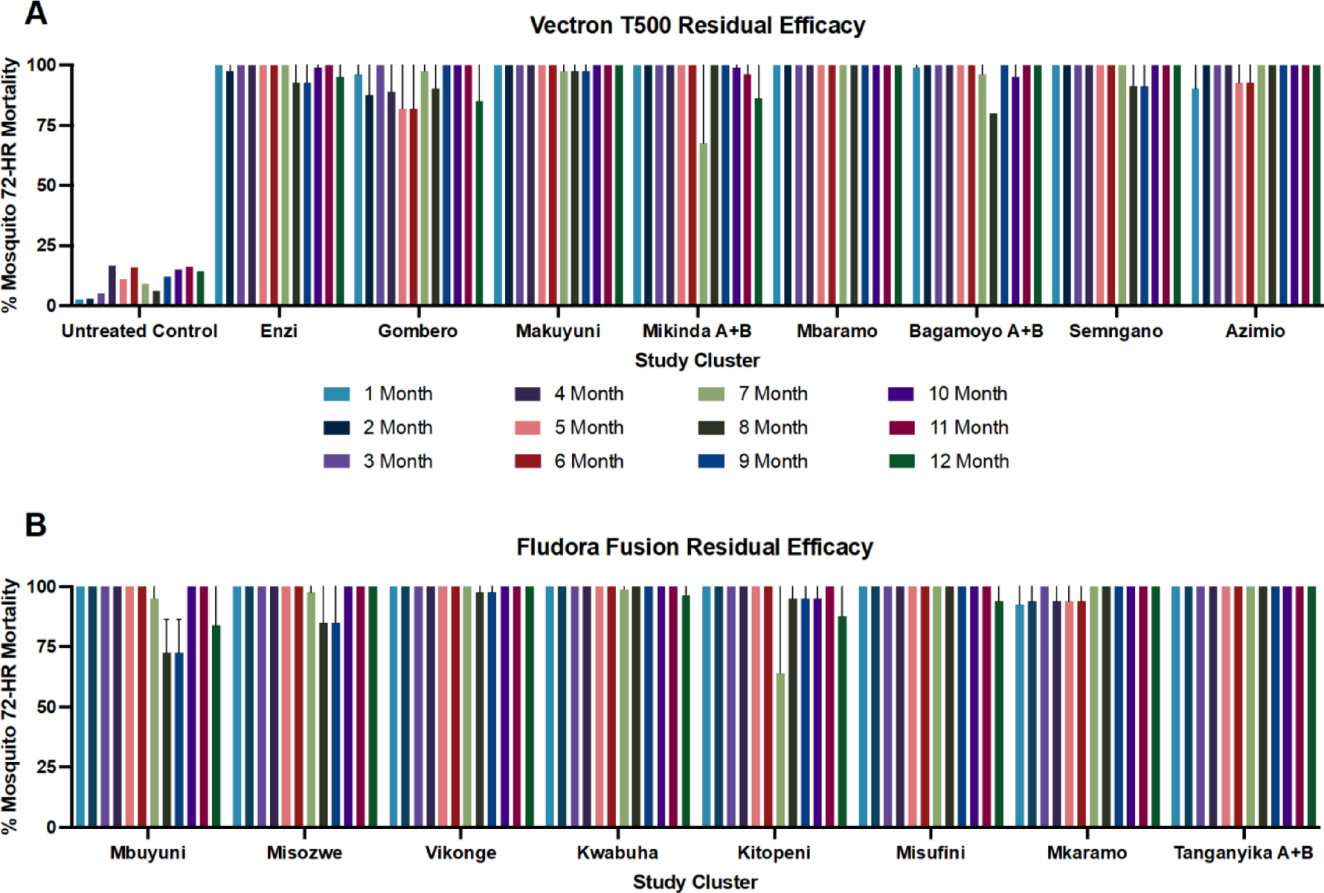
VECTRON ™ T500 (**A**) and Fludora^®^ Fusion (**B**) clusters post – spraying wall cone bioassays results against susceptible *An. gambiae* s.s. Kisumu (months 1 to 5 and 7 to 10) and resistant *An. gambiae* s.s. Muleba-Kis strains (months 6, 11 and 12).

A total of 12 monthly rounds of in situ wall cone bioassays with pyrethroid-susceptible and pyrethroid-resistant strain mosquitoes were conducted. In most cone bioassays conducted, both VECTRON™ T500 and Fludora^®^ Fusion induced over 90% mortality of susceptible *An. gambiae* s.s. Kisumu strain and resistant *An. gambiae* s.s. Muleba-Kis strain mosquitoes on both concrete and mud walls up to 12 months after spraying (Fig. 1). With both VECTRON™ T500 and Fludora^®^ Fusion, relatively higher mortalities were recorded on concrete walls than on mud walls. The residual efficacy for both interventions 12 months post spraying remained above 80% for both concrete and mud walls (Fig. 1).

### Vector bionomics and insecticide resistance monitoring

The insecticide susceptibility testing results indicated that *An. gambiae* s.l. from Muheza was resistant to deltamethrin (mortality rates < 90%) (Supplementary Fig. 1).

A total of 7,127 mosquitoes were collected from the 16 study clusters during the baseline period between January 2021 and the second week of February 2021. Of these 3,262 (46%) were morphologically identified as non-malaria vectors (*Culex* species) and 3865 were *Anopheles* mosquitoes. Of the *Anopheles* mosquitoes, 2357 (32%) and 1488 (21%) were morphologically identified as *An. gambiae* s.l. and *An. funestus* s.l., respectively. The other 41 specimens (1%) were identified as other Anophelines.

**Fig. 2:**
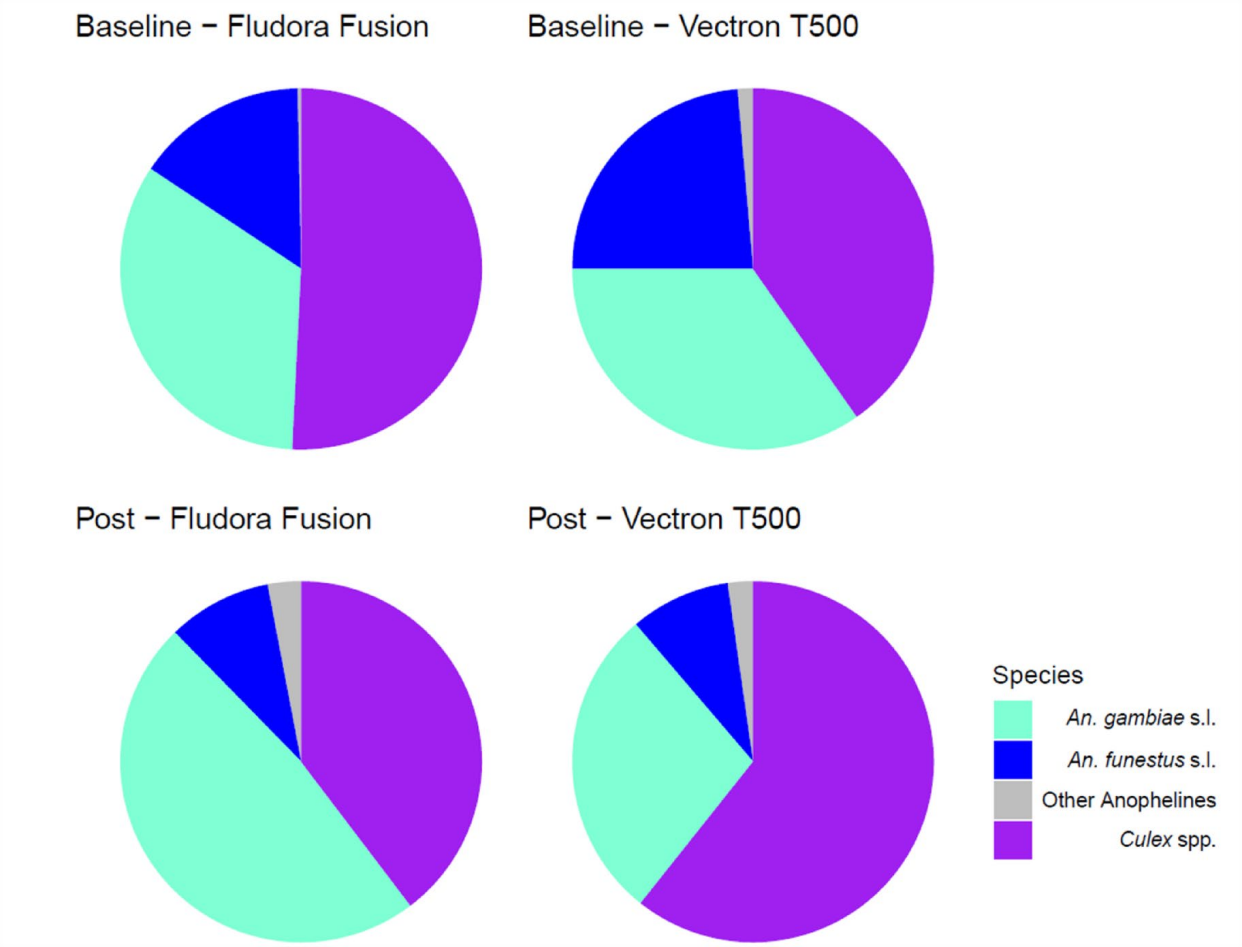
Proportional species composition of mosquitoes sampled from the VECTRON™ T500 and Fludora^®^ Fusion clusters at baseline and post intervention.

**Fig. 3:**
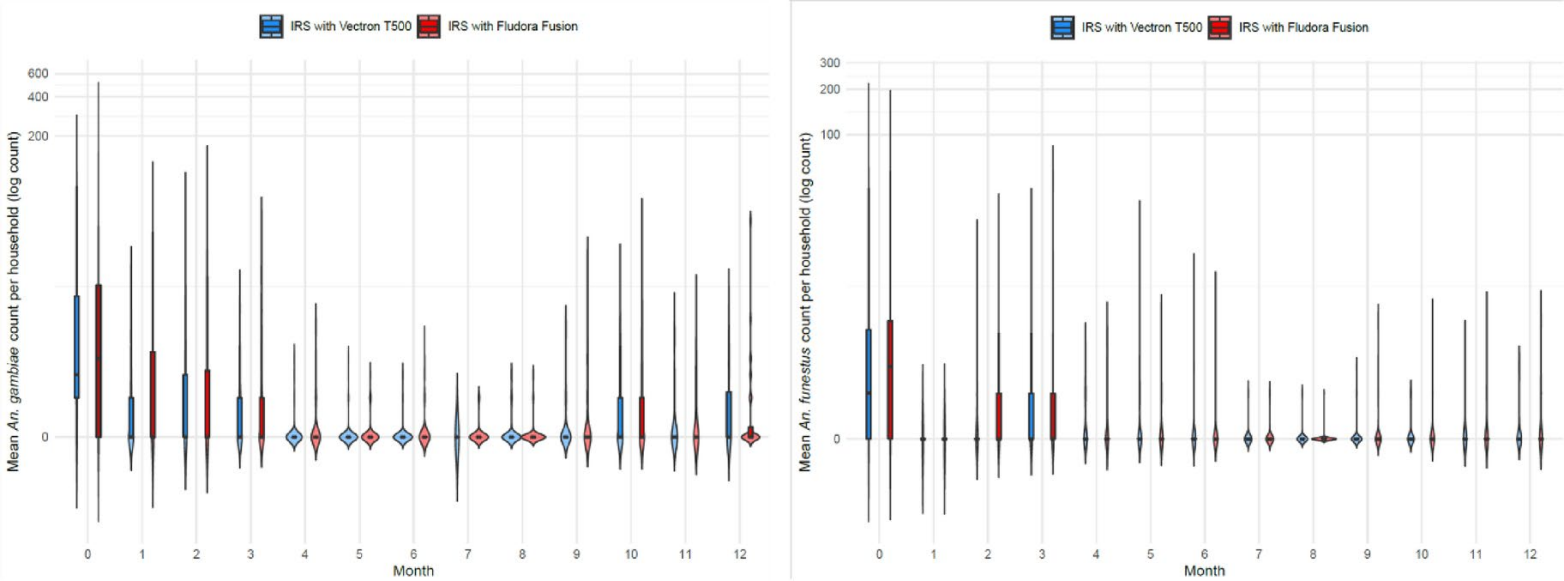
Monthly violin plots showing *An. gambiae* s.l. and *An. funestus* mosquito counts per household across treatment arms. The violin plots illustrate the density distribution of mosquito counts per house, while the boxplots inside each violin show the interquartile range (IQR), with the horizontal line representing the median count per household. The y-axis represents the mean count of *An. gambiae* (top) and *An. funestus* (bottom), log-transformed (*log(1 + x)*) to account for highly skewed mosquito count distribution.

**Fig. 4:**
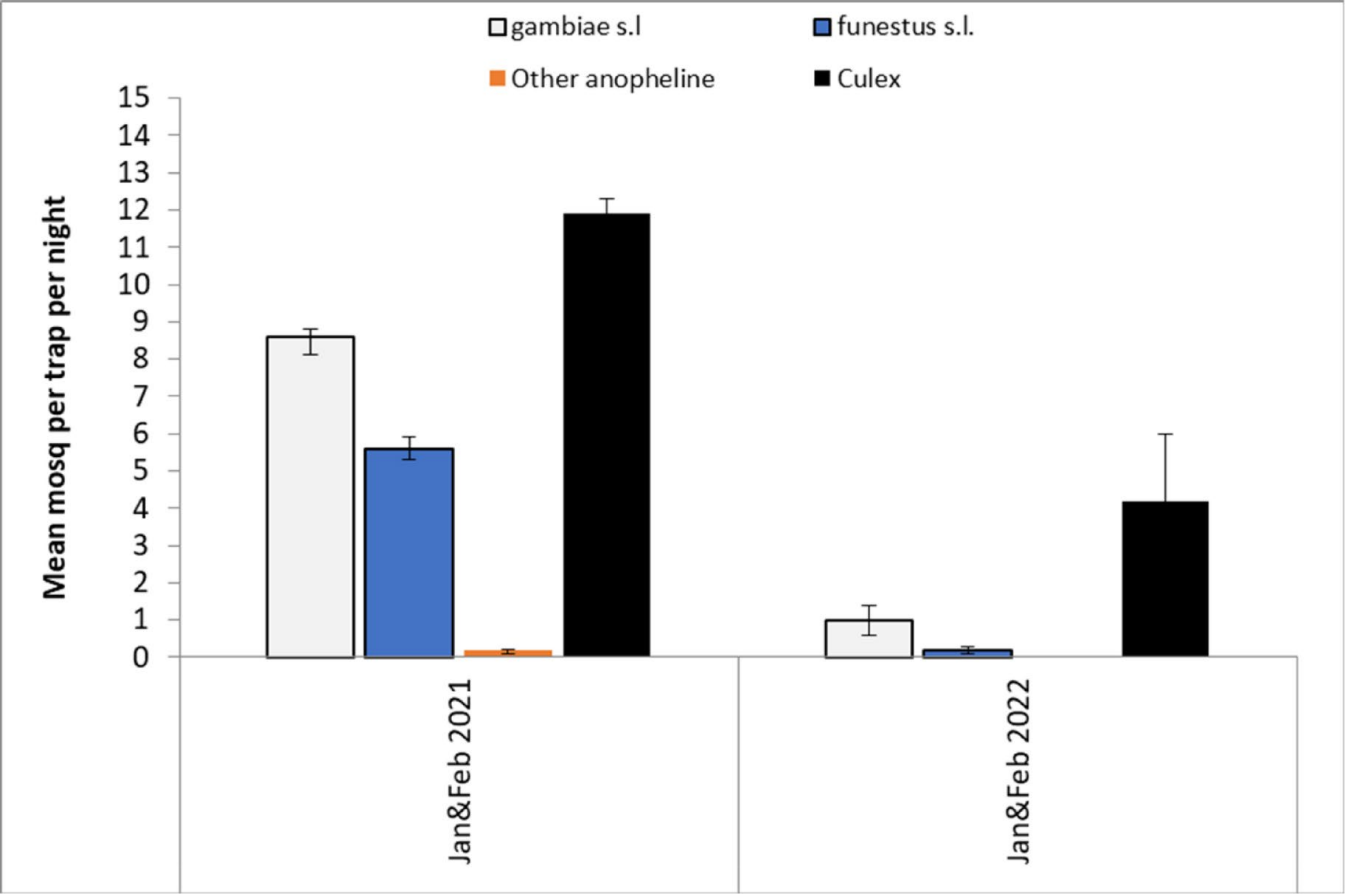
Comparison of the mean number mosquitoes caught in CDC light traps per species complex between the pre-intervention (January – February 2021) period and those caught during the same two months post-intervention in 2022.

A sequence of 12 rounds of monthly CDC light trapping was carried out in 5 randomly selected houses per cluster. Proportionally, species of the *Culex* genus were most abundant, followed by *An. gambiae* s.l. and then *An. funestus* s.l. (Fig. 2). The density of *An. gambiae* s.l. was considerably higher than *An. funestus* s.l. As expected from its biology, *An. funestus* s.l. was a late season vector due to its proclivity for breeding in more permanent bodies of water compared to *An. gambiae* s.l. which, if predominantly *An. gambiae s.s.* would prefer temporary breeding sites. Both *An. gambiae* s.l and *An. funestus* recorded a declining trend in abundance post intervention (Fig. 3). All species declined significantly from the baseline survey to the first month post survey (*P* = 0.001). With all surveys, the number of Anopheline mosquitoes collected from the VECTRON™ T500 arm were not significantly different to those collected from the Fludora^®^ Fusion arm (Table 1). The prolonged impact of both IRS products over one year is illustrated in Fig. 4.

For the pre-intervention period, the results showed that the average number of indoor Anopheline mosquitoes collected per house per night in Fludora^®^ Fusion clusters was 17.4, (95% CI: 0.9–33.9), while the average number of indoor Anopheline mosquitoes collected in VECTRON™ T500 clusters was 15.4 (95% CI: 0.5–30.3). The difference between the two arms pre-intervention was not significant (Table 1). Post-intervention, the results showed that the average number of indoor Anopheline mosquitoes collected per house per night decreased: in Fludora^®^ Fusion clusters it was 1.6 (95% CI: 0.2–3.0), while the average number of indoor Anopheline mosquitoes collected post-intervention in VECTRON ^TM^ T500 clusters was 1.5 (95% CI: 0.03–2.9). The difference in vector density between VECTRON™ T500 arm and Fludora^®^ Fusion arm post intervention was not significant (Table 1).

**Table 1:** Anopheline mosquito density pre and post intervention in the VECTRON™ T500 and Fludora^®^ Fusion trial arms.

Trial arm		Mean number per house per night (95% CI)	Incidence rate ratio (95% CI)	*P* value
Pre-intervention	Fludora^®^ Fusion	17.4 (0.86–33.9)	1 (Ref)	
	VECTRON™ T500	15.4 (0.50–30.3)	0.89 (0.27–2.86)	0.84
Post-intervention	Fludora^®^ Fusion	1.6 (0.2–3.0)	1 (Ref)	
	VECTRON™ T500	1.5 (0.03–2.9)	0.92 (0.36–2.35)	0.858

The mean geometric village level density of indoor Anopheline mosquitoes collected per house per night in the Fludora^®^ Fusion treated clusters was 2.87 while the mean geometric village level density of indoor Anopheline mosquitoes collected in the VECTRON™ T500 treated clusters was 2.32. The total *Anopheles* density ratio was 0.77 (95% CI: 0.45–1.29), after adjustment for baseline density, month of catch, percentage of houses with ITNs and percentage of houses plastered with mud (Table 2). The mosquito density ratios according to taxon are shown in Table 2.

**Table 2:** Overall mosquito densities in each trial arm and ratio between trial arms.

Species	VECTRON™ T500 geometric mean village level density (mosquitoes per house per night)	Fludora^®^ Fusion geometric mean village level density (mosquitoes per house per night)	Ratio (95% CI)	Adjusted ratio* (95% CI)
Total *Anopheles*	2.32	2.87	0.81 (0.46, 1.41)	0.77 (0.45, 1.29)
*An. gambiae*	1.33	1.97	0.68 (0.33, 1.40)	0.65 (0.32, 1.29)
*An. funestus*	0.80	0.77	1.05 (0.52, 2.12)	0.98 (0.50, 1.93)
Other *Anopheles*	0.05	0.05	0.90 (0.35, 2.30)	0.86 (0.35, 2.14)
*Culex*	1.56	2.55	0.61 (0.25, 1.49)	0.59 (0.24, 1.47)

*Adjusted for month of catch, baseline density, % of houses with ITNs and % of mud houses.

The species identification assays revealed that the majority of Anopheline samples were *An. gambiae* s.s., being between 60% and 67% in both trial arms before and after spraying (Table 3). The species composition of samples assayed before and after spraying are highlighted in Table 3.

**Table 3:** Species composition of anopheline mosquitoes from the VECTRON™ T500 and Fludora^®^ fusion trial arms pre- and post-intervention.

	Spp	Pre-intervention		Post-intervention	
		n/N	Percentage, (95%CI)	n/N	Percentage, (95%CI)
VECTRON™ T500	Ga	517/859	60.2, (56.8–63.4)	370/600	61.6 (57.6–65.6)
	Ar	64/859	7.5, (5.7–9.4)	82/600	13.6 (11.0–16.7)
	Fu	277/859	32.2, (29.1–35.5)	129/600	21.5 (18.3–25.0)
	Lees	0/859	0, (0–4.3)	5/600	0.83 (0.8–1.9)
	Par	0/859	0, (0–4.3)	3/600	0.5 (0.1–0.5)
	Riv	1/859	0.12, (0.0–0.6)	11/600	1.8 (0.9–3.3)
Fludora^®^ Fusion	Ga	881/1366	64.5 (61.9–67.0)	768/1162	66.1 (63.3–68.8)
	Ar	172/1366	12.6 (10.9–14.5)	194/1162	16.7 (14.6–19.0)
	Fu	308/1366	22.5 (20.4–24.9)	181/1162	15.6 (13.5–17.8)
	Lees	3/1366	0.2 (0.00–0.6)	2/1162	0.1 (0.0–6.2)
	Par	0/1366	0 (0–2.7)	0/1162	0 (0–3.2)
	Riv	2/1366	0.1 (0.0–0.5)	17/1162	1.5 (0.85–2.3)

Ga = *An gambiae* s.s. Ar = *An. arabiensis*, Fu = *An. funestus* s.s. Lees = *An. leesoni*, Par = *An. parensis*, Riv = *An. rivulorum.* n = total in each species, N = total number of Anopheline mosquitoes.

The mean village level *P. falciparum* sporozoite rates in the VECTRON™ T500 and Fludora^®^ Fusion trial arms were 0.39% and 1.04%, respectively (Table 4). The difference, adjusted for baseline measure, month of catch, percent of houses with ITNs and percent of houses with mud plastered walls, was 0.84% (95% CI: −1.24–2.93%). The mean village level EIR in the VECTRON™ T500 and Fludora^®^ Fusion trial arms were 3.18 and 15.44, respectively (Table 4). The difference adjusted for baseline measure, month of catch, percent of houses with ITNs and percent of mud plastered houses was 15.61 (95% CI: −11.56–42.79).

**Table 4:** Sporozoite and entomological inoculation rates in each trial arm.

Measure	VECTRON™ T500 village level mean (*n* = 1686)	Fludora^®^ Fusion village level mean (*n* = 2871)	Difference (95% CI)	Adjusted difference* (95% CI)
Sporozoite rate	0.39%	1.04%	0.65% (1.69%, −0.38%)	0.84% (2.93%, −1.24%)
EIR (infectious bites per person per year)	3.18	15.44	12.26% (27.87, −3.35)	15.61% (42.79, −11.56)

*Adjusted for month of catch, baseline measure, % of houses with ITNs and % of mud houses; EIR is entomological inoculation rate.

A total of 1,341 *An. gambiae* s.s. and *An. arabiensis* were analysed for the *kdr* mutation L1014S. Of these, 812 and 529 were collected at baseline and after IRS, respectively. Molecular analysis for *kdr* in *An. gambiae* s.s. and *An. arabiensis* samples showed a significant difference in the frequency of homozygous resistant (RR), heterozygous resistant (RS), and homozygous susceptible (SS) after IRS compared to the baseline in the Fludora^®^ Fusion arm (X^2^ = 27.77; *P* < 0.001; Table 5). Similarly, in the VECTRON™ T500 arm, there was a significant difference in the frequency of RR, RS and SS after IRS compared to baseline (X^2^ = 14.39; *P* = 0.001; Table 5). The frequency of RR, RS and SS pre- and post-intervention for each species are shown in Supplementary Tables 1 and 2.

**Table 5:** Kdr (L1014S) target site mutation frequency before and after IRS in each arm.

	Before intervention		After intervention	
	Fludora^®^ Fusion No (%; 95%CI)	VECTRON™ T500 No (%; 95%CI)	Fludora^®^ Fusion No (%; 95%CI)	VECTRON™ T500 No (%; 95%CI)
Homozygous resistant (RR)	284 (43; 39–47)	75 (48; 40–56)	204 (60; 54–65)	88 (47; 40–54)
Heterozygous resistant (RS)	242 (37; 33–41)	61 (39; 31–47)	103 (30; 25–35)	47 (25; 19–32)
Homozygous susceptible (SS)	130 (20; 17–23)	20 (13; 8–19)	35 (10; 7–14)	52 (28; 21–35)
Total	656 (100)	156 (100)	342 (100)	187 (100)
*kdr frequency*	810 (62; 59–64)	211 (68; 62–73)	511 (75; 71–78)	223 (60; 54–65)
Total	1,312 (100)	312 (100)	684 (100)	374 (100)

### Adverse effects and acceptability

Participants in all groups reported that the spray exercise was organized in such a way that it did not interfere with any of their routine household activities. It was reported that before the spray team arrived at a consented house, one person visited the targeted houses a few hours prior to the spray team to remind house owners about house preparations for spraying. According to participants, the reminder also gave them the opportunity to shift some of the in-house activities such as cooking to outside the house. This served to minimise the interference of the spraying on household activities.

*“There is someone who passed earlier to check if the properties in the house have been arranged properly*,* then later on the sprayer came for the spraying*,* therefore*,* they found us we have already taken out all properties…….”* (Male, p6, Muungano).

Another female participant added that;

*“The activities indeed were neither stopped nor affected*,* for us who resides in the house since there were early pass of the information*,* hence making all issues such as preparing of children food was conducted earlier before the arrival of the team at the house…….”* (Female, p4, Misozwe).

All group discussants in intervention and reference clusters expressed a preference for village residents to serve as spray operators. They noted that, having known the sprayers for years, it would not be likely for them to disclose household secrets, as the community shares living conditions. Consequently, revealing another household’s secrets would be regarded the same as revealing their own. In addition, discussants highlighted that the spraying activity provided income for their children and youths, which contributed to the village development. Finally, some participants mentioned that using sprayers from within the village enhanced security, as their behaviour was known and trusted, unlike that of strangers.

*“I think the inside village sprayers should continue since they already knew our life in the house*,* it is easy for them to keep our secrets….”*(Female, p5, Misozwe).

All group discussants reported initially having great interest and high expectations for the spray exercise, believing that the intervention could completely eliminate mosquitoes and malaria in the village. In all the intervention clusters, group discussants reported a tremendous decline in mosquito density, similar to group discussants from the Fludora^®^ Fusion clusters, where discussants noted that mosquitoes disappeared in the first three weeks after spraying. They continued by adding that after the first three weeks of the spray, mosquitoes returned into the houses. Opinions varied on why mosquitoes returned; some discussants believed it was due to standing water and bushes which surround their houses. In one male group from the intervention cluster, it was suggested that the product which was sprayed in their village was fake, since they did not observe any changes in the mosquito abundance.

*“Since we were told that there would be no mosquitoes we expected to see no mosquitoes indeed*,* and frankly speaking at first there were no mosquitoes at all*,* but in the coming days say after three weeks the mosquitoes returned back….”* (Male, p6, Mtindiro).

All discussants showed interest in having their entire house sprayed. In some clusters, house owners decided which room should be sprayed or given priority, but in other clusters the sprayers were the one who made these decisions. For the clusters where homeowners chose, the whole house was sprayed. However, in other clusters in which the sprayers decided, some of the preferred rooms were not sprayed. A few discussants, especially in the female groups, commented that toilets/bathrooms and kitchen should not be left unsprayed to ensure comprehensive protection from mosquito bites, especially as the bathrooms were marked as areas with a lot of resting mosquitoes.

*“I also prefer the spray to be sprayed even in the toilets and bathrooms as well as other surrounding places*,* because if you go in the toilets mosquitoes can bite you there*,* because there are so many mosquitoes there*,* they can miss you in the seating room but they will get you in the toilets….”*(Female, p8, Misozwe).

Most did not experience or hear of any adverse events after the spray campaign. A few women in the intervention arm reported having experienced an increased heartbeat, unpleasant smells and skin itching. Minor reactions were noted by others which included physical fatigue, burning face sensation, dry throat and dizziness. Some discussants admitted entering their houses before the recommended waiting period after spraying, accidentally coming into contact with the chemicals, or sleeping on wet sprayed beds. These mild reactions typically lasted only a few hours and claimed were alleviated by washing with water and drinking milk. No medical attention was required for these adverse events.

*“I passed near the window during the spraying and I was accidentally sprayed on the face*,* at that time I felt normal but later on I started feeling face itching*,* hence I decided to wash by water but after few minutes of washing the face my throat became dried and started feeling some fire burning in the stomach….”* (Female, p6, Mtindiro).

*“…….there after we were told to wait outside the house for two hours*,* but after they left I decided to go inside and take some of my two oranges*,* I picked them washed and peel*,* after peeling I ate them*,* after eating them I started feeling dizzy and loss of bodily strength….”* (Female, p9, Misozwe).

## Discussion

For the first time since the Millenium there has been an upsurge of malaria in parts of sub-Saharan Africa. Over the last 20 years there has been a steady increase in ITN coverage of various classes, but the last decade has also seen a gradual decrease in IRS coverage^13^. If Africa is to eliminate malaria, the international community should revise its malaria vector control policy and consider combining dual interventions of different classes of ITNs and IRS in areas where malaria transmission remains persistently high and resources are sufficient. There has been only one formal community randomised trial of Dual-AI ITNs and long-lasting IRS – that of a PBO ITNs combined with pirimiphos-methyl IRS (Actellic^®^300 CS) in a malaria hot spot in North West Tanzania^14^. These were the only long-lasting IRS and ITN products approved by WHO and available at the time. Regrettably, there was a negative interaction between the PBO on the net and the mosquito cytochrome P450 enzymes responsible for the activation of the pro-insecticide pirimiphos-methyl to the active metabolite in the pyrethroid resistant vector population^14^. Hence the mixture of PBO net and pirimiphos-methyl IRS failed to be additive in terms of reducing vector density and this has set back any further evaluations of the possible benefit of dual ITN plus IRS interventions.

VECTRON™ T500 effectively controls vectors which have target site resistance mechanisms such as *kdr* found in *An. gambiae* or metabolic upregulated P450-based resistance mechanisms found in *An. funestus*, and therefore would be appropriate for combining with new classes of ITN. According to WHO for a new candidate IRS product to be listed in the IRS intervention class, it must demonstrate non-inferiority to an IRS product which has already demonstrated public health value (Fludora^®^ Fusion) in a community (Phase III) cluster randomized indoor residual spraying trial^15,16^.

In this trial, VECTRON™ T500 was non-inferior to Fludora^®^ Fusion in terms of its ability to reduce the vector density, sporozoite rate, entomological inoculation rate, and malaria transmission for one year.

In terms of residual efficacy, at twelve months post spraying, both VECTRON™ T500 and Fludora^®^Fusion remained effective based on cone bioassay mortality criteria which remained above the 80% threshold against insecticide susceptible and pyrethroid resistant mosquitoes. Although efficacy was relatively higher on concrete walls compared to mud walls, the differences were not significant. Implementing IRS with long-lasting insecticide formulations that have residual activity and can reduce vector density throughout the year is essential in reducing the overall cost of IRS, which is a key challenge to universal coverage of any IRS intervention. The long-lasting residual efficacy of at least 12 months of the candidate VECTRON™ T500 and reference Fludora Fusion present an important development to the IRS intervention class. In Benin the residual efficacy of VECTRON™ T500 as shown in an experimental hut trial and in a two‑arm non‑inferiority community randomised evaluation of VECTRON™ T500, the residual efficacy was up to 18 months and up to 24 months, respectively^12,13^. However, it should be noted that in Benin, the local practice when using mud to plaster houses is to mix the mud with cement, which is different to the practice in Tanzania where only mud plaster is used.

Molecular assays for Anopheline species identification revealed that the majority of vectors were from the *An. gambiae* complex (*An. gambiae s.s.* and *An. arabiensis*) with the minority from the *An. funestus* complex (*An. funestus s.s.*,* An. parensis*,* An. leesoni* and *An. rivulorum*). Where *An. gambiae s.s.* and *An. arabiensis* are sympatric in Tanzania, such as around Lake Victoria, the sporozoite rate of *An. arabiensis* is lower than that of *An. gambiae*s.s. and its zoophilic/exophilic tendencies are greater^17–19^. Less is known about the vector status of sibling species of the *An. funestus* complex; *An funestus s.l.*has increased in frequency in North Tanzania^20,21^. Considering that all CDC light traps were conducted inside houses, this could be an indication of the importance of some of these sibling species as malaria vectors in the study area. 

The increased frequency of *kdr-*VGSC RR and RS genotype in the Fludora^®^ Fusion arm could be an indication of further pyrethroid selection in this arm. Conversely, the small increase *kdr-*VGSC SS genotype frequency in VECTRON™ T500 arm may indicate a restoration of pyrethroid susceptibility in the *An. gambiae* s.l. populations. In a community trial conducted in Benin, there was no significant change in *kdr* frequency in either VECTRON™ T500 or Fludora^®^Fusion arms^13^.

In conclusion, the impact of the VECTRON™ T500 IRS intervention on vector density and EIR was non-inferior to that recorded for the WHO reference product, Fludora^®^Fusion, thereby fulfilling the WHO criterion for an IRS product. Secondly, its long residual efficacy of up to 12 months post spraying makes it more cost effective when compared to many other WHO approved IRS products. Study findings provide important operational information for National Malaria Control and regional malaria elimination programmes to utilise when designing prospective, pragmatic insecticide resistance management schemes and regional elimination programmes. This supports the strategy of rotation IRS products containing insecticides with different modes of action for insecticide resistance management in combination with standard ITN or dual-active ingredient ITNs^17^. To further enhance malaria control gains, there is a need for new IRS products to be safe to use, and additive in effect to combine with PBO ITNs. VECTRON™ T500 and Fludora^®^ Fusion with their novel modes of action and, unlike pirimiphos-methyl IRS, not requiring metabolic activation, could be used as IRS partners in a rotation strategy to regain control of malaria in Africa.

## Methodology

### Study area

This study was carried out in Muheza District, Tanga Region, Northeast Tanzania^22^. The population of Muheza District consists primarily of subsistence farmers^23^. The region has a tropical climate with a long rainy season from March to June and a short rainy season from October to December. Most houses within Muheza District are constructed from mud or concrete with a thatched or iron roof. In 2014, surveys conducted in Muheza District found that 83% of households owned at least one ITN, and there have been two universal ITN distribution campaigns since this date^24^. Muheza District had received nets from the targeted mass replacement campaign, which took place about a month before spraying for this study was carried out. Therefore, the majority (80%) of the ITNs were Olyset Plus and the average household coverage was 87% (Supplementary Table 4). Malaria transmission occurs all year round with two peaks in transmission following the two rainy seasons. The main malaria vectors in Muheza District are *An. gambiae* s.s., *An. arabiensis* and *An. funestus*s.s^24^. Wild *An. gambiae*s.l. in Muheza District are resistant to pyrethroids^25^.

### Study design

This study was a mixed method design that employed both quantitative and qualitative methods. The study protocol was published earlier^22^. The quantitative component utilized a matched cluster randomized controlled non-inferiority study design, appropriate for evaluating IRS as a community-level intervention. The study monitoring period was for 12 months post-intervention, between April 2021 and March 2022. Baseline data collection on vector densities was conducted in January and February 2021. At the onset of the long rainy season in March 2021, one round of IRS was completed, which was followed by monthly vector density monitoring and in situ wall cone bioassays. The primary trial outcome was Anopheline indoor vector density post-intervention, adjusted for baseline density, month of collection, proportion of houses with ITNs and proportion of mud houses. The secondary outcomes were: (1) EIR, an indicator of malaria transmission, defined as the average number of sporozoite-positive mosquitoes per night per household, and weighted to account for the proportion of collected *Anopheles* mosquitoes processed for analysis; (2) sporozoite rate, defined as the proportion of *Anopheles* mosquitoes found to be infected with *Plasmodium falciparum*; and (3) intervention residual bio-efficacy. The qualitative component of the trial included a social-cultural and acceptability study. Baseline household enumeration, along with social-cultural and acceptability assessments, were conducted prior to the IRS intervention. After the IRS campaign, these factors were monitored and compared to baseline data^22^.

### Power calculation, estimation of effect size and cluster selection

The sample size calculation was based on the study primary endpoint i.e., indoor density of *Anopheles* mosquitoes. An estimated mean of 3.0 female *Anopheles*mosquitoes per light trap per night was used, referencing data from recent studies^14^. A paired design was used in which clusters were matched on the baseline indoor density of *Anopheles*mosquitoes. Previous data suggested a within-pair coefficient of variation of 0.23^26^, but a more conservative value of 0.3 was used in power calculations for this study.

The trial was designed to demonstrate that spraying the intervention product, VECTRON™ T500, would not result in higher vector densities, by a prespecified non-inferiority margin of 50%, than those per trap per night in the reference arm sprayed with Fludora^®^Fusion^22^. Using the methods described in Hayes and Moulton^26^ and the assumptions listed above, 8 clusters per treatment arm (16 in total) were needed for the 50% margin to have 80% power to demonstrate non-inferiority. The calculations were performed based on 49 trap nights per cluster. The number of clusters considered the loss of degrees of freedom caused by matching.

**Fig. 5:**
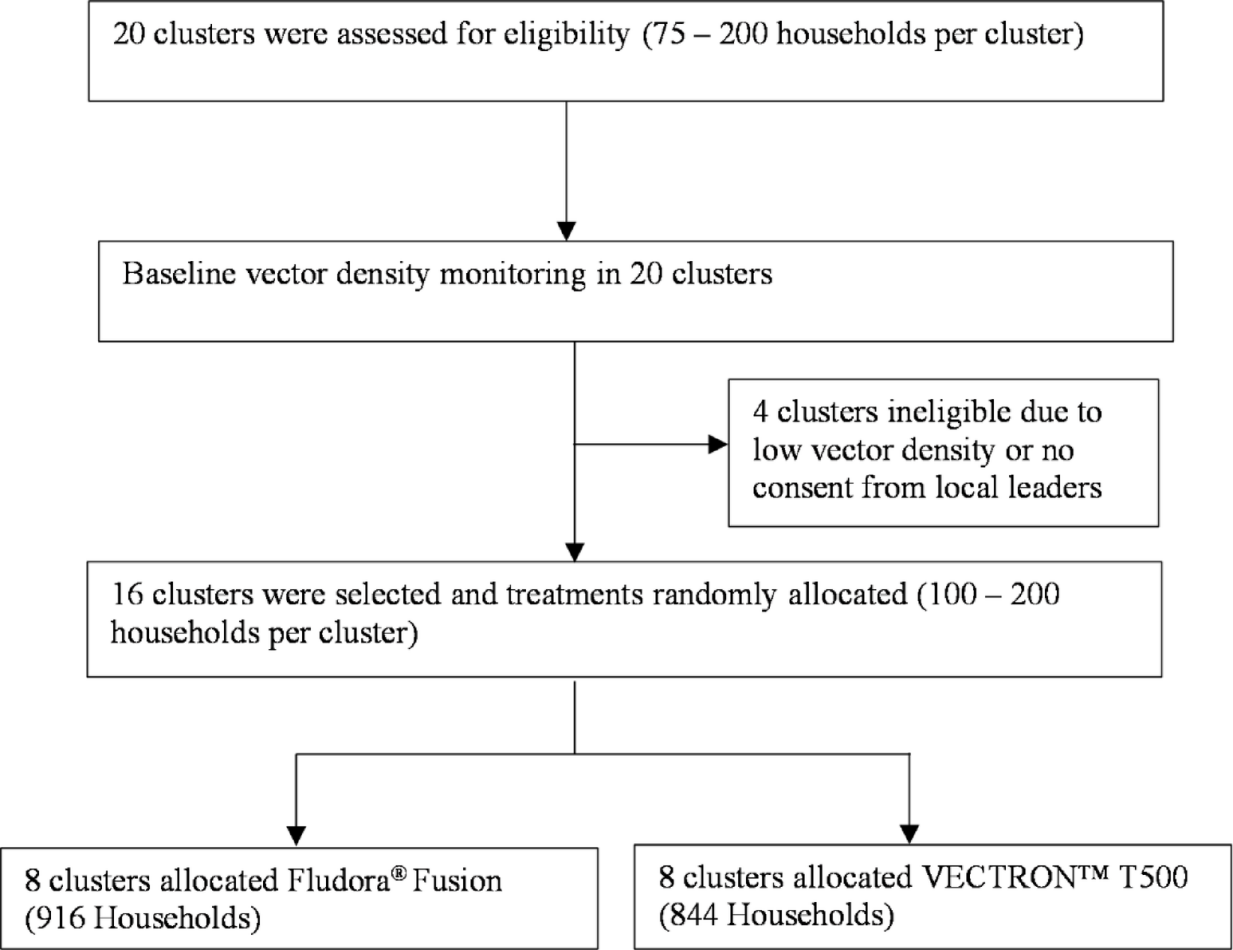
Summary of study design.

Sixteen clusters, containing 75–200 households per cluster, were selected for spraying. Core areas of clusters were at least 1 km apart to prevent infiltration of mosquitoes from other villages^22^. During baseline vector density monitoring, 20 clusters were included to enable adequate pair-matching of the 16 clusters needed to power the study (Fig. 5). Four clusters were dropped or ineligible based on different reasons including organic farming that was been practised in or near the two clusters, extremely low vector density that was observed in one cluster and reluctancy to participate in the study in one cluster. The 16 selected clusters were pair-matched based on vector densities and randomly allocated to the two trial arms: one arm (8 clusters) was sprayed with the novel intervention VECTRON™ T500 and the other arm (8 clusters) with the reference product Fludora^®^ Fusion. All possible randomisations were stratified on ITN coverage and wall surface type to ensure a minimum difference of 15% or less in mean ITN coverage and mean ratio of concrete houses to mud houses per arm.

### Procedures

Fifty-six 11 L Hudson^®^X-pert compression sprayers (H.D Hudson Manufacturing Company, Foreman St. Lowel, U.S.), each fitted with a 1.5 bar control flow valve and an 8002E nozzle were used. Calibration tests were done three times by filling each sprayer with 7.5 L of water and pressurising it to 40 psi. The target spray discharge was 550 mL per minute according to the WHO recommendations^27^.

Consenting households in the intervention arm were sprayed with VECTRON™ T500 (100 mg/m^2^ broflanilide) and consenting households in the reference arm received IRS with Fludora^®^ Fusion (200 mg/m^2^ clothianidin and 25 mg/m^2^deltamethrin). A total of 100 spray operators were recruited from the clusters and trained according to the WHO IRS operational manual by a company from South Africa which had many years of experience with IRS in the national MCP^27^. Spray operators were provided with personal protective equipment and clothing, and health checks were performed on them before and after spraying. All sleeping and living rooms in houses were sprayed, whilst kitchens and storerooms were excluded.

Whatman^®^ No. 1 filter papers were attached to the walls of households before spraying and then removed post-spraying for chemical analysis at the Liverpool School of Tropical Medicine, UK. The results of the chemical analysis will be reported elsewhere.

WHO cone bioassays on IRS treated walls of houses were used to assess residual activity in both trial arms at monthly intervals for 12 months post spraying^22,28^. Four households were randomly selected from each cluster for monthly monitoring of residual efficacy using the wall-cone bioassay. Following exposure, mosquitoes were placed in paper cups and monitored for up to 72 h to assess mortality at 24, 48 and 72 h. WHO cones were affixed to four walls per house in a diagonal arrangement across the walls, and one additional cone was affixed to an outside untreated wall to act as a control. Of the four selected households per cluster, the walls of two were constructed out of concrete and the other two out of mud, to compare residual activity on these substrate materials. To determine IRS product bioefficacy, both insecticide susceptible (*An. gambiae* s.s. Kisumu) and pyrethroid resistant (*An. gambiae*s.s. Muleba-kis) strains were used^7,8^. The Muleba-Kis strain was resistant to pyrethroids based on the *kdr* (L1014S) target site mutation and a metabolic mechanism associated with overexpression of the cytochrome P450-dependent monooxygenase CYP6P3^29^. The strain was susceptible to carbamates and organophosphates and did not express the *Ace-1*mutation^7,29^.

In each cone bioassay test, 10 *An. gambiae*s.s. adult, unfed, 2–5-day old female mosquitoes^28^ were aspirated into each cone and exposed for 30 min. After exposure, mosquitoes were held in paper cups with access to cotton wool soaked in 10% glucose solution. Immediate knockdown and delayed mortality at 24, 48 and 72-hours post-exposure was recorded.

The WHO/CDC bottle bioassays were used to determine any phenotypic resistance of wild *Anopheles* mosquitoes to broflanilide (6 µg/bottle), deltamethrin (12.5 µg/bottle) and clothianidin (90 µg/bottle without Mero^®^), according to WHO/CDC methods. The above concentrations of these insecticides have been shown to kill 100% of insecticide susceptible mosquitoes^13,30,31^. The broflanilide bottle bioassay dosage was determined by conducting dose response bioassays with the insecticide susceptible *An. gambiae* s.s. Kisumu strain. Discriminating concentration determination was undertaken using BioRssay in RStudio v4.0.2. The preliminary discriminating concentration for use in the broflanilide susceptibility monitoring associated with this community trial was defined as twice the LC_99_at 72 h post exposure as stipulated by WHO Control of Neglected Tropical Diseases (NTD) and WHO Global Malaria Programme (GMP)^32^.

Broflanilide bottles were coated mixed with the adjuvant Mero^®^(Bayer Crop Science) at 800 ppm. Insecticide solutions were prepared from technical grade material dissolved in acetone^33^.

*An. gambiae* s.l. were collected as larvae from breeding sites within the study area and reared in the insectary. Progeny of these wild *An. gambiae*s.l. were then exposed to insecticide according to standard operating procedures (using insecticide-coated Wheaton bottles)^31^. Two-to-five-day old, unfed female mosquitoes were exposed in bottles for 1 h. Knockdown was recorded for all insecticides after 1 h. Mortality was recorded after 24 h for deltamethrin, and at 48 and 72 h for broflanilide and clothianidin. Assays were conducted with wild *An. gambiae* s.l. at baseline and 3 months after IRS. CDC/WHO bottle bioassays were also performed with *An. gambiae*s.s. Kisumu (insecticide susceptible strain) from lab colonies for comparison and quality control^16^. *An. gambiae* Kisumu is a standard susceptible laboratory strain originally colonised in 1953 from Kenya that is widely used in Africa. Muleba-kis is a cross between Kisumu and a wild pyrethroid resistant strain colonised in Muleba, Kagera, and pressurised using permethrin every generation to maintain pyrethroid resistance. The strain is widely used in the PAMVERC laboratories in Tanzania.

CDC light traps, hung inside houses next to occupied ITNs, were used to collect mosquitoes for species identification^18^ and *P. falciparum*circumsporozoite protein enzyme linked immunosorbent assay (CSP-ELISA)^19,34^. Four operatives were allocated with 5 light traps each and given responsibility for trapping in 4 of the 16 clusters. In each cluster each month, there were 3 rounds of CDC light trap collections spaced over 3 weeks in 15 randomly selected households. Therefore, in each treatment arm of 8 clusters there were 15 light trap nights in each month. All households in each cluster were re-randomised at each monthly surveillance point. Therefore, in each cluster there were 180 light-trap nights spread across the 12-month post intervention period, from which non-inferiority was determined. All mosquitoes collected were identified morphologically to genus and species complex level (*An. gambiae* s.l., *An. funestus* s.l. and *Culex*spp.). Sub-samples taken from each cluster each month were molecular identified to species level using Taqman real-time PCR assays^18^; numbers and proportions identified depended on the total numbers caught; *P. falciparum*CSP-ELISA was performed on all molecular identified Anopheline species^22^. A subsample of molecularly identified *An. gambiae* s.s. and *An. arabiensis* were subjected to Taqman real-time PCR assays to detect the *kdr*target site mutation (L1014S)^35^.

### Adverse effects and acceptability assessment

The social-cultural and acceptability study was conducted in four of the sixteen sprayed clusters, selected at random, that is, two clusters each in the intervention and reference study arms. The selected clusters in the VECTRON™ T500 intervention arm were the villages Muungano and Pangamlima whilst in the Fludora^®^ Fusion arm Misozwe and Mtindiro were selected. Eight community focus group discussions (FGDs), two in each cluster were conducted, with 63 study participants.

### Statistical analysis

Data analysis was carried out using cluster level summaries^22^, using Stata (Version 16) since random effects models perform poorly in a matched design with fewer than 20 clusters per arm^26^. For the primary endpoint of mosquito density, the ratio of densities in each matched pair was calculated and inference was based on a paired *t*-test. Adjustments were made for time post-spraying, ITN use and wall type using the two-stage method described by Hayes and Moulton^26^. The secondary outcomes of *P. falciparum* sporozoite rate and estimated number of infective bites per person per year (EIR) were analysed in the same way.

## Electronic supplementary material

Below is the link to the electronic supplementary material.

Supplementary Material 1

## Data Availability

All data associated with this study are present in the paper or the appendix. All other relevant data are available from the corresponding author upon reasonable request.
