# Supplementary material for: A noninferiority cluster randomised evaluation of a broflanilide indoor residual spraying insecticide, VECTRON T500, for malaria vector control in Tanzania
Source: Sci Rep. 2025 Apr 29;15:15013. doi: 10.1038/s41598-025-99809-9 (PMC12041253; doi:10.1038/s41598-025-99809-9)
Supplement: Supplementary file 1 — Supplementary Material 1 [file 41598_2025_99809_MOESM1_ESM.docx]

**A non-inferiority cluster randomised community** **evaluation of VECTRON^TM^ T500 (broflanilide WP) compared to Fludora^®^ Fusion (clothianidin-deltamethrin WP-SB) for malaria vector control by indoor residual spraying in Tanzania**

Appendix

**Table of Contents**

[Supplementary Figure 1: Insecticide susceptibility test results on wild field sampled *An. gambiae s.l.* done at baseline and after 3 months post survey 1](#_Toc192193417)

[Supplementary Figure 2: Study area A location of Muheza District in Northeast Tanzania; B clusters treated with either VECTRON™ T500 or Fludora^®^ Fusion in Muheza District. 2](#_Toc192193418)

[Supplementary Table 1. *Kdr* (L1014S) target site mutation frequency before and after IRS in each arm for *An. arabiensis*. 3](#_Toc192193419)

[Supplementary Table 2. *Kdr* (L1014S) target site mutation frequency before and after IRS in each arm for *An. gambiae* s.s. 4](#_Toc192193420)

[Supplementary Table 3: Averages of basic demographic information per trial arm. 4](#_Toc192193421)

[Supplementary Table 4: Basic information per cluster and trial arm assigned. 5](#_Toc192193422)


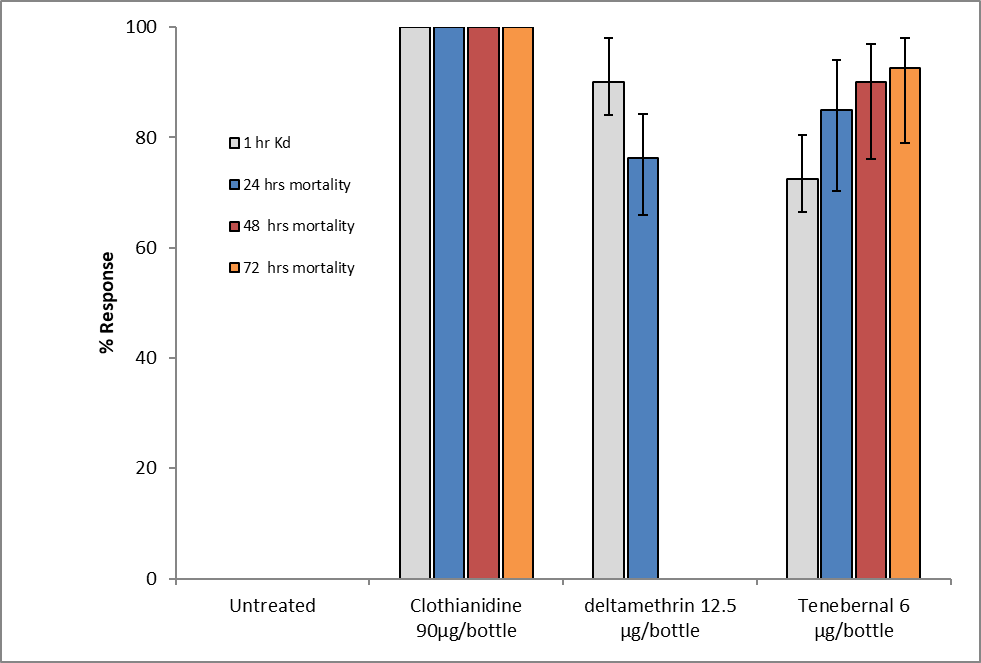


# Supplementary Figure 1: Insecticide susceptibility test results on wild field sampled *An. gambiae s.l.* done at baseline and after 3 months post survey


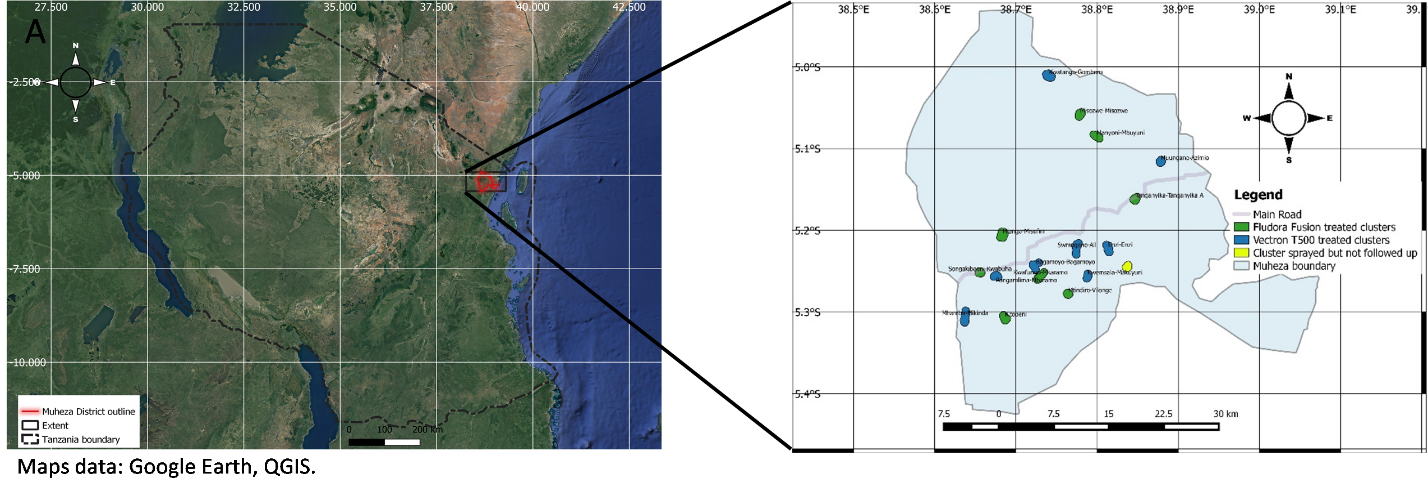


# Supplementary Figure 2: Study area A location of Muheza District in Northeast Tanzania; B clusters treated with either VECTRON™ T500 or Fludora^®^ Fusion in Muheza District.

The maps of Tanzania and Muheza district were produced using QGIS 3.22 software and overlaid on Google Earth satellite images by the study investigator (NJM). Using QGIS 3.22 software, a one kilometre transect from the mid-point to the perimeter of each village was digitized to produce the shape files for the clusters in the Muheza map

# Supplementary Table 1. *Kdr* (L1014S) target site mutation frequency before and after IRS in each arm for *An. arabiensis*.

|  | Fludora® Fusion | | VECTRON™ T500 | |
| --- | --- | --- | --- | --- |
|  | Pre-intervention  No (%; 95%CI) | Post-intervention  No (%; 95%CI) | Pre-intervention  No (%; 95%CI) | Post-intervention  No (%; 95%CI) |
| Homozygous resistant (RR) | 12 (10; 5 – 18) | 0 (0) | 0 (0 | 1 (2; 0 – 11) |
| Heterozygous resistant (RS) | 0 (0) | 0 (0) | 0 (0 | 0 (0) |
| Homozygous susceptible (SS) | 103 (90; 82 – 94) | 21 (100) | 14 (100) | 47 (98; 89 – 100) |
| Total | 115 (100) | 21 (100) | 14 (100) | 48 (100) |
| X^2^, P-value | 2.40, 0.121 | | 0.30, 0.586 | |
| *kdr frequency* | 24 (10; 7 – 15) | 0 (0) | 0 (0) | 2 (2; 0 – 7) |
| Total | 230 (100) | 42 (100) | 28 (100) | 96 (100) |

# Supplementary Table 2. *Kdr* (L1014S) target site mutation frequency before and after IRS in each arm for *An. gambiae* s.s.

|  | Fludora® Fusion | | VECTRON™ T500 | |
| --- | --- | --- | --- | --- |
|  | Pre-intervention  No (%; 95%CI) | Post-intervention  No (%; 95%CI) | Pre-intervention  No (%; 95%CI) | Post-intervention  No (%; 95%CI) |
| Homozygous resistant (RR) | 284 (52; 48 – 57) | 204 (64; 58 – 69) | 75 (53; 44 – 61) | 87 (62; 54 – 71) |
| Heterozygous resistant (RS) | 230 (42; 38 – 47) | 103 (32; 27 – 37) | 61 (43; 35 – 52) | 47 (34; 26 – 42) |
| Homozygous susceptible (SS) | 27 (5; 3 – 7) | 14 (4; 2 – 7) | 6 (4; 2 – 9) | 5 (4; 1 – 8) |
| Total | 541 (100) | 321 (100) | 142 (100) | 139 (100) |
| X^2^, P-value | 10.19, 0.006 | | 2.76, 0.251 | |
| *kdr frequency* | 798 (74; 71 – 76) | 511 (80; 76 – 83) | 211 (74; 69 – 79) | 221 (79; 74 – 84) |
| Total | 1,082 (100) | 642 (100) | 284 (100) | 278 (100) |

# Supplementary Table 3: Averages of basic demographic information per trial arm.

| **Row Labels** | **Average of % mud** | **Average of mean An** | **Average of % LN coverage** | **Average of no. of houses** |
| --- | --- | --- | --- | --- |
| FF | 80.75 | 15.1875 | 85.0125 | 142.75 |
| V | 77.625 | 15.1375 | 90.825 | 154.625 |
| **Grand Total** | **79.1875** | **15.1625** | **87.91875** | **148.6875** |
| **Difference** | **-3.87%** | **-0.33%** | **6.84%** | **8.32%** |

% is percentage; FF is Fludora^®^ Fusion and V is VECTRON™ T500

# Supplementary Table 4: Basic information per cluster and trial arm assigned.

| **Cluster** | **No. of houses** | **% mud houses** | **mean Anopheles density** | **% LN coverage** | **Trial arm** |
| --- | --- | --- | --- | --- | --- |
| Kwafungo-Mkaramo | 179 | 77 | 53.9 | 71 | FF |
| Bagamoyo-Bagamoyo B | 116 | 81 | 51 | 65.1 | V |
| Mianga-Misufini | 130 | 69 | 22.1 | 68.6 | FF |
| Kwemsala-Makuyuni | 200 | 72 | 21 | 97.4 | V |
| Semngano-all | 165 | 66 | 19.4 | 93.7 | V |
| Mtindiro-Vikonge | 137 | 79 | 14.2 | 100 | FF |
| Mhamba-Mikinda A+B | 142 | 91 | 13.6 | 97.9 | V |
| Songa Kibaoni-Kwabuha | 150 | 85 | 13 | 75.9 | FF |
| Pangamlima-Mbaramo | 174 | 90 | 10.5 | 93.4 | V |
| Manyoni-Mbuyuni | 100 | 81 | 9.8 | 86.8 | FF |
| Kitopeni-all | 181 | 87 | 6.6 | 77.8 | FF |
| Kwatango-Gombero | 160 | 92 | 2.7 | 96 | V |
| Enzi-Enzi | 130 | 80 | 2.5 | 100 | V |
| Misozwe-Misozwe | 160 | 91 | 1.7 | 92 | FF |
| Muungano-Azimio | 150 | 49 | 0.4 | 83.1 | V |
| Tanganyika-Tanganyika A | 105 | 77 | 0.2 | 98 | FF |

% is percentage; FF is Fludora^®^ Fusion and V is VECTRON™ T500
